# Designing a multi-epitopic vaccine against the enterotoxigenic *Bacteroides fragilis* based on immunoinformatics approach

**DOI:** 10.1038/s41598-019-55613-w

**Published:** 2019-12-24

**Authors:** Mahnoor Majid, Saadia Andleeb

**Affiliations:** Department of Industrial Biotechnology, Atta-ur-Rahman School of Applied Biosciences (ASAB), National University of Sciences & Technology (NUST), Islamabad, 44000 Pakistan

**Keywords:** Clinical microbiology, Pathogens

## Abstract

Enterotoxigenic *Bacteroides fragilis* is an enteric pathogen which is described as a causative agent of various intestinal infections and inflammatory diseases. Moreover, various research studies have reported it to be a leading factor in the development of colorectal cancer. As a part of the normal human microbiome, its treatment has become quite a challenge due to the alarming resistance against the available antibiotics. Although, this particular strain of *B. fragilis* shows susceptibility to few antibiotics, it is pertinent to devise an effective vaccine strategy for its elimination. There is no vaccine available against this pathogen up to date; therefore, we systematically ventured the outer membrane toxin producing proteins found exclusively in the toxigenic *B. fragilis* through the *in-silico* approaches to predict a multi-epitopic chimeric vaccine construct. The designed protein constitutes of epitopes which are predicted for linear B cells, Helper and T cells of outer membrane proteins expected to be putative vaccine candidates. The finalized proteins are only expressed in the enterotoxigenic *B. fragilis*, thus proving them to be exclusive. The 3D structure of the protein was first predicted followed by its refinement and validation via utilizing the bioinformatic approaches. Docking of the designed protein with the TLR2 receptor forecasted apt binding. Upon immune simulation, notable levels were observed in the expression of the immune cells.

## Introduction

Human microbiome is a large reservoir of trillions of microbial species which have co-evolved with the human bodies. Since from the beginning of mankind, these microbes have been creating selective ecosystems which depend on the specific habitats while staying acclimatized to the physiology of the hosts^1^. Such microorganisms which form a stable community in the intestinal tracts of human are regarded as crucial regulators of immune and metabolic hemostasis. Moreover, the gut microbiome also serves as a moderator of resistance to particular pathogenic infections^2^. Based on multiple research studies, it has been concluded that the species of *Homo sapiens* is more microbial as per the cell numbers which constitutes about 3.8·10^13^ of bacterial cells and 3.0·10^13^ of human cells^3^. Any disruption in the normal microbial population is regarded as dysbiosis which is defined as the alterations in the composition of the inhabitant commensal population that resides in the heathy individuals^4^. As the human microbiome has found to exhibit a significant position in the development of host processes, it is observed that such changes in its composition might serve as a major factor in the occurrence and sustenance of various pathological conditions^5^. Microbial structure in the human gut can be influenced by various determinants such as diet, genetic makeup of the host, seasonal infections and medical interventions for instance, the frequent and inappropriate usage of antibiotics. The alternating use of antibiotics is a prime reason for predisposing the population to immune diseases^6^.

Out of various species that reside in the human gut, Bacteroidetes is a highly dominating phylum representing ten to twenty percent of the total microbial population present in the colon^7^. Members of this phylum are highly important as they are involved in a number of significant mechanisms. This lineage of bacteria was one of the earliest ones to emerge as a result of the evolutionary processes. Bacteria belonging to this particular group are anaerobic, non-spore formers, Gram negative rods and resistant to bile^8^. *B. fragilis* is the most compelling member of the Bacteroidetes phylum as it is multi-faceted having commensal roles which can flip into an opportunistic pathogen. Moreover, it is the most frequent anaerobic isolate identified in clinical specimens therefore, reported as a virulent species^9^. This bacterial species is further differentiated into two categories according to their capability of secreting a toxin known as *B. fragilis* toxin that is a metalloprotease dependent on zinc. The strains which produce this toxin are termed as enterotoxigenic *B. fragilis* (ETBF) while the non-secreting class is known as nontoxigenic *B. fragilis* (NTBF)^10^. The enterotoxigenic *B. fragilis* is a major causative agent of diarrhea and inflammation in the intestines which in some cases, leads to the development of colorectal cancer. The fragilysin toxin produced by this strain of *B. fragilis* has the ability to cleave the E-cadherin that is attached to the β-catenins intracellularly. As a result of this cleavage, the amount of free catenins in the cytosol increases which becomes a prime factor in the transcription of *c-myc* oncogenes that are a part of T-cell factors. A number of studies have confirmed the fragilysin toxin being the first bacterial toxin that has the ability to activate the Tcf dependent β-catenin nuclear signaling, thus making *B. fragilis* a potent contributor of oncogenic transformations in the intestinal cells^11^.

Colorectal cancer refers to the undefined division of cells in the colon or rectum (colorectum) which is a main part of the human gastrointestinal tract. On the number scale, it ranks as third most recurring diagnosed cancer in males and second most in females^12^. Based on the data collected by the World Health Organization (WHO), almost 1, 849, 518 recent cases of colorectal cancer were reported in people of all ages while the death toll was reported to be 880,792 globally in 2018^13^. Moreover, the regional incidence of colorectal cancer varies 10-fold where Australia and New Zealand are the top-rated countries which have highest registered rates of incidence and mortality followed by Europe and North America. Contrarily, the lowest rates of incidence and mortality were observed in South-Central Asia and Africa^13,14^. A number of features that are involved in the onset of the disease and variations have been reported based on the age groups of the patients. Owing to the economic burden and the serious health concerns which colorectal cancer creates to the public, a number of control programs have been initiated to manage this precarious case. Center for Disease Control and Prevention started its Colorectal Cancer Control Program (CRCCP) in 2005. The basic goal of this program is to effectively monitor the population suffering from colorectal cancer and provide a direct screening service. As, the risk of developing this cancer becomes higher in ages of 50 to 75, therefore the program focuses on providing screening service to the people who have crossed the ages of 50^15^.

There are a number of screening programs for the management of colorectal cancer but the main driver responsible for its pathogenesis is still neglected. The effective management of Enterotoxigenic *B. fragilis* in the human gut is essential so as to minimize the stressed environment that leads towards the occurrence of the disease. Antibiotics are the main source of treating this strain of *B. fragilis* but, due to the phenomenon of antibiotic resistance, those drugs which were once given to treat the resulting infections have now lost their efficacy. Ciprofloxacin, erythromycin, ampicillin, tetracycline have found to be resistant against the *B. fragilis* group in most of the cases^16^. By considering all these factors, it has become crucial to devise new therapeutic strategies to tackle the life-threatening conditions associated with the toxigenic *B. fragilis*.

It has been proven by various studies that Gram negative bacteria makes use of the type III, type IV and type VI secretions systems that transport the virulence factors to the host however, genetic analyses of the toxigenic *B. fragilis* does not indicate the presence of these systems rather it revealed a T6SS loci. T6SS is a multi-protein complex which is divided into three distinct loci named as GA1, GA2 and GA3^17^. The GA3 loci is specifically present in *B. fragilis* which is regarded as a source of novel immunity proteins and effectors. Howbeit, this locus is not found to be involved in the secretion of toxin from the *B. fragilis*. A large number of enzymes consisting of outer membrane vesicles (OMVs) are formed by the enterotoxigenic *B. fragilis* which indicate the importance of these OMVs in the export processes^18^.

This work was carried out to design a prophylactic vaccine by employing *in silico* approaches. According to a well-established study, enterotoxigenic *B. fragilis* consists of a pathogenicity island known as BfPA1 that is encompassed within the conjugative transposon CTn86. The study further reports the presence of exclusive toxin producing proteins associated with the pathogenicity island which are most likely to be delivered to the hosts through the OMVs. The focus of this research plan was the creation of a potent multi-epitopic vaccine candidate against the toxigenic *B. fragilis* by employing the unique proteins i.e. fragilysin toxin protein and metalloprotease II protein found only in the toxigenic strains of the target bacteria^19^.

## Results

### Collection of protein sequences and introductory analysis

The sequences of the selected proteins were retrieved from NCBI database for the construction of a multi-epitopic vaccine against the toxigenic *B. fragilis*. The two proteins used for this study WP_005797262.1 and WP_005797263.1 were selected based on their exclusive presence in the enterotoxigenic *B. fragilis*. SignalP 4.1 predicted the functional sequences of the proteins succeeding the splitting of the signal peptide. According to the results, the proteins have secretory signal peptides. DeepLoc and Vaxign predicted the localization of the proteins according to which both of the proteins are localized in the extracellular compartment. The protein sequences were then further subjected to the epitope prediction of B, T and Helper cells. The *Lactobacillus rhamnosus* GG 50 s ribosomal protein L2 (AXI95322.1) was collected from the UniProt database and employed as an adjuvant in the vaccine model due to the reported adjuvant properties of the probiotic strain and its ability to interact with TLR2 receptor^20^. Adjuvant is an essential part in a vaccine formulation as it tends to amplify its potency^21^.

### Linear B-cell epitopes identification

Various online servers were used for the forecast of B cell epitopes but, only the recurrent epitopes were selected for the building up of chimeric vaccine. BepiPred 2.0 and BCPred predicted such epitopes simultaneously, thus being incorporated in the multi-epitopic vaccine design **(**Table 1**)**.

**Table 1:** Proteins and epitopes conservation in related *Bacteroides*.

Protein	B-cell Epitopes	CTL Epitopes	HTL Epitopes
WP_005797262.1	(1) VLYTTEYSCPSGNADEGLDG (0%)	(9) MQDAANSVY (0%)	(5) FILNFNKMKNVKLLL (0%)
	(2) EADSLTTSIDTPVTASIDLQ (0%)	(10) GMSTTQLMY (0%)	(6) ILNFNKMKNVKLLLM (0%)
WP_005797263.1	(3) SDKIVVCNTGEDTRSGNSDI (0%)	(11) STTSSSHPY (0%)	(7) KNSILSLSSRATYPA (0%)
	(4) PRGNFEVAAISTTSSSHPYT (0%)	(12) RMAEIANYY (0%)	(8) KTFGYASGIGVIHLN (0%)

**Figure 1:**
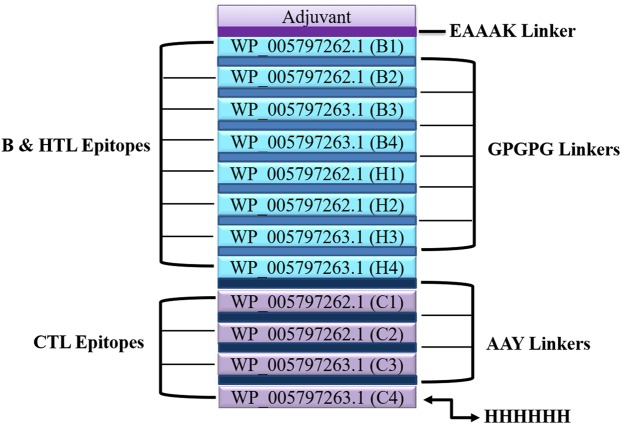
Schematic presentation of the final multi-epitope vaccine protein. The 512-amino acid long protein sequence containing an adjuvant (light purple) at the amino terminal end linked with the multi-epitope sequence through an EAAAK linker (purple). B-cell epitopes and HTL epitopes are linked using GPGPG linkers (blue) while the CTL epitopes are linked with the help of AAY linkers (dark blue). A 6x-His tag is added at the Carboxy terminus for purification and identification purposes.

Predicted linear B-cell epitopes and T-cell epitopes selected in order to design the vaccine protein and their percentage of amino acid identity among the non-toxigenic *Bacteroides fragilis* strains is in brackets. The serial numbers assigned to the epitopes indicate the order of positions in the final design of the chimera in Fig. 1.

### T-Cell epitopes (CTL) estimation

NetCTL 1.2 server predicted a sum of 35 CTL (9-mer) ligands for the two finalized proteins. Default parameters were opted for the prediction of epitopes out of which 04 epitopes were finalized based upon their high binding scores **(**Table 1**)**.

### Helper T lymphocytes (HTL) prediction

NetMHCII 2.2 web server predicted the MHC-II epitopes with the highest binding corresponding to the alleles from human i.e. HLA-DR, HLA-DQ and HLA-DP based on the IC_50_ scores. A total of 04 HTL epitope were chosen for the ultimate chimeric construct **(**Table 1**)**.

### Conservation of proteins and epitopes in related *Bacteroides*

A comprehensive BLAST analysis of the specified proteins against the UniProt database disclosed that the outer membrane proteins were found to be highly conserved in the Enterotoxigenic *B. fragilis* as these are the toxin producing proteins which are absent in the other nontoxigenic strains of *B. fragilis* and species of *Bacteroides* respectively **(**Tables 2, 3). The enterotoxigenic *B. fragilis* was found to exclusively home the predicted proteins according to the multiple sequence alignment of the identified B and T cell epitopes. Tables 1–3 overall show the conservation of the proteins in other *Bacteroides* along with the percentage identities of the finalized epitopes.

**Table 2:** Shared identity of the selected proteins in Nontoxigenic commensal strains.

Protein	Percentage Identity in Nontoxigenic *B. fragilis* Strains		
	*B. fragilis* YCH46	*B. fragilis* 638R	*B. fragilis* NCTC 9343
WP_005797262.1	Not found	Not found	Not found
WP_005797263.1	Not found	Not found	Not found

**Table 3:** Shared identity of the selected proteins in related *Bacteroides* species.

Protein	Percentage Identity in Related *Bacteroides* Species				
	*B. distasonis*	*B. ovatus*	*B. thetaiotamicron*	*B. vulgatus*	*B. uniformis*
WP_005797262.1	Not found	Not found	Not found	Not found	Not found
WP_005797263.1	Not found	Not found	Not found	Not found	Not found

### Assemblage of multi-epitopic subunit vaccine candidate

A total of 04 linear B-cell epitopes, 04 CTL epitopes and 04 HTL epitopes were used for the construction of multi-epitopic vaccine chimera. The vaccine was built by adding the adjuvant which was a L2 ribosomal protein with accession no. AXI95322.1 to the amino (N) terminus of the peptide sequence and attached to the first B-cell epitope through an EAAAK linker in order to prompt a specific immune response. Further, the B-cell and HTL epitopes were linked together via GPGPG linkers whereas for connecting the CTL epitopes, AAY linkers were utilized. At the C-terminus of the vaccine sequence, a 6xHis tag was incorporated for protein identification and purification purposes. The final chimeric construct constituted 512 amino acids with a molecular weight of 54 kDa **(**Fig. 1**)**.

### Antigenicity and allergenicity evaluation of the vaccine protein

VaxiJen 2.0 web server predicted the antigenicity of the vaccine design attached with an adjuvant, to be 0.765 with the bacterial model and 0.622 with a virus model by opting a threshold of 0.4. ANTIGENpro predicted the antigenicity score to be 0.933909. The antigenicity of the vaccine candidate was also checked without including the adjuvant part for which VaxiJen gave scores of 0.7326 in a model of microbe and 0.4933 in a model of virus, at a threshold of 0.4. These results indicate that the vaccine protein sequence, either linked with an adjuvant or not, is antigenic in nature. AllerTOP v.2 and AllergenFP online servers predicted the vaccine sequence to be non-allergenic in nature in the presence and absence of the adjuvant.

### Analysis of solubility and physiochemical properties

ExPASY ProtParam predicted the molecular weight (MW) of the specified vaccine protein to be 54 kDa. The pI (Theoretical isoelectric point value) of the protein was calculated to be 9.75. According to this value, the protein is considered as highly basic in nature. The half-life of the subjected protein was determined to be 30 hours in mammalian reticulocytes *in vitro*, >20 hours in yeast and >10 hours in *E. coli in vivo*. Furthermore, based on the estimation of PROSO II, the protein was found to be soluble in its expression with a solubility score of 0.558. The instability index (II) of 35.64 was predicted for the protein by ProtParam, ranking it as a stable model as values greater than 40 indicate instability. The aliphatic index of the protein was estimated to be 72.27 which confirms its thermostability^22^. GRAVY, the grand average of hydropathicity for the protein was predicted to be −0.5. The negative value indicates that the protein is hydrophilic in nature and can easily interact with the water molecules^23^.

### Secondary structure extrapolation

**Figure 2:**
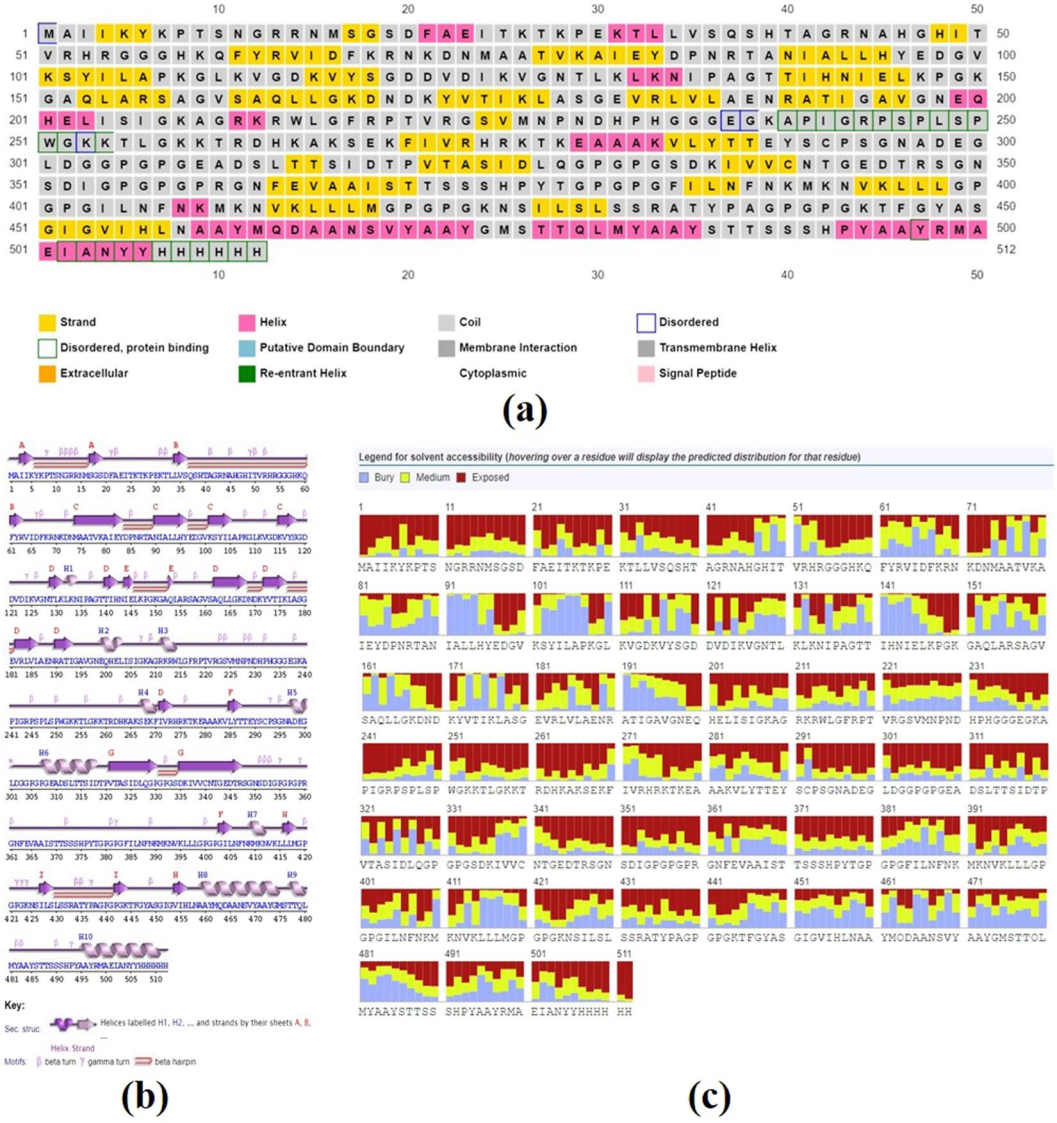
Graphical representation of secondary structure features of the final subunit vaccine sequence. (**a,b**) The protein is predicted to comprise helices (8.0%), beta strands (25.0%) and coils (66.0%) (**c**) Based on the accessibility of the amino acid residues, 52% were predicted to be exposed, 21% medium exposed and 25% were predicted to be buried in the designed protein.

RaptorX generated the secondary structure of the chimeric protein and the results indicated that the protein constitutes 8% helix, 27% beta strand and 66% coil (Fig. 2a,b**)**. Besides this, 52% were predicted to be exposed, 21% medium exposed and 25% were predicted to be buried based on the accessibility of the amino acid residues **(**Fig. 2c**)**. A total of 11% residues were found to be localized in the disordered domains.

### Tertiary structure assessment of the protein

**Figure 3:**
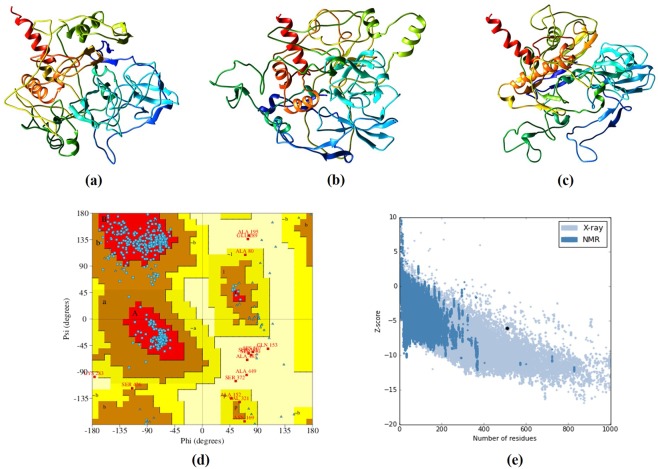
Protein modelling, refinement and validation. (**a**) The final 3D model of the multi-epitope vaccine obtained after homology modelling on I-TASSER. (**b**) Refined model obtained via ModeRefiner (**c**) The refined 3D structure by GalaxyRefine. Validation of the refined model with (**d**) Ramachandran plot analysis showing 93.7%, 4.5% and 1.8% of protein residues in favoured, allowed, and disallowed (outlier) regions respectively and (**e**) ProSA-web, giving a Z-score of −6.04.

Five tertiary structure models of the chimeric construct were predicted by the I-TASSER server by employing 10 threading templates. Out of these models, 3i1nC, 5czpA, 5czpY, 3j3v and 6hmaC were the best ones. The 10 selected templates have good alignment according to their Z-score values that span from 1.78 to 2.86. The five models that are provided by the server have their C-score values varying from −1.85 to −3.22. The standard C-score value usually ranges between −5 and 2, where the positive value indicates more confidence. In this study, the highest C-score model, derived from the homology modelling was picked for future refinement protocol **(**Fig. 3a–c**)**. The results indicate a predicted TM score of 0.991 and RMSD value of 0.784 ± 3.7 Å. For analyzing the similarities between two protein structures, the TM score is assessed which dissolve all the fluctuations related to the RMSD values. A model with a TM score greater than 0.5, shows accurate topology whereas, a model with a TM score less than 0.17 indicates non-specific similarity^24^.

### Refinement of the tertiary structure

Initially the unprocessed chimeric vaccine model was refined by ModRefiner after which the resulting structure was further refined on the GalaxyRefine that provided five models. Out of these models, model 1 was chosen based on multiple parameters such as GDT-HA (0.9932), RMSD (0.254) and MolProbity (1.989). The clash score was calculated to be 10.7, score of poor rotamers was 0.2 and Ramachandran plot predicted a score of 93.1%. This model was thus finally picked as the chimeric model for later investigations.

### Validation of the model stability

The Ramachandran plot analysis of the protein model predicted that 93.7% of the residues of the refined protein model are present in the favored regions. This score is steady with the 93.1% score obtained through the GalaxyRefine procedure. Moreover, 4.5% of the residues were found to be present in the allowed regions and only 1.8% in the disallowed or outlier boundary **(**Fig. 3d**)**. ProSA-web and ERRAT server authenticated the overall quality and the occurrence of errors that might potentially arise in the refined model. The refined model under study, was considered to be appropriate as it exhibits 87% quality factor using ERRAT and a Z-score of −6.04 with ProSA-web **(**Fig. 3e**)**.

### Prediction of discontinuous B-Cell epitopes

**Figure 4:**
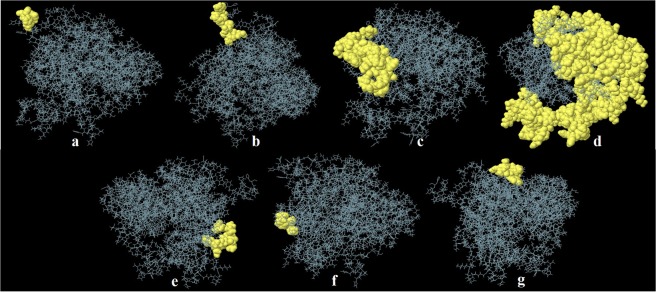
Discontinuous B-cell epitopes predicted by the ElliPro. (**a–g**) Three-dimensional representation of conformational or discontinuous epitopes of the highest antigenic chimeric protein of Enterotoxigenic *Bacteroides fragilis*. The epitopes are represented by yellow surface, and the bulk of the protein is represented in grey sticks.

The prediction of seven discontinuous B cell epitopes revealed the presence of 290 total residues in them with scores varying from 0.529 to 0.977. The size of these epitopes was found within the range of three to two hundred and twenty-six residues **(**Table 4, Fig. 4**)**.

**Table 4:** Discontinuous B-cell epitopes predicted by the ElliPro.

No.	Residues	Number of residues	Score
1	A:H508, A:H509, A:H512	3	0.977
2	A:A500, A:E501, A:I502, A:A503, A:N504, A:Y506, A:H507, A:H510, A:H511	9	0.79
3	A:R261, A:D262, A:H263, A:K264, A:A265, A:K266, A:S267, A:E268, A:K269, A:F270, A:I271, A:V272, A:R273, A:A367, A:I368, A:S369, A:T370, A:T371, A:S372, A:S373, A:S374, A:H375, A:P376, A:Y377, A:T378, A:G379, A:P380, A:G381, A:P382, A:G383, A:I385, A:L386, A:N387	33	0.696
4	A:K7, A:P8, A:T9, A:S10, A:N11, A:G12, A:R13, A:R14, A:N15, A:M16, A:A22, A:I24, A:T25, A:K26, A:T27, A:K28, A:P29, A:E30, A:K31, A:T32, A:L33, A:L34, A:V35, A:S36, A:Q37, A:S38, A:H39, A:T40, A:A41, A:G42, A:R43, A:N44, A:A45, A:H46, A:G47, A:H48, A:I49, A:T50, A:V51, A:R52, A:H53, A:R54, A:G55, A:G56, A:G57, A:H58, A:K59, A:Q60, A:F61, A:V64, A:I65, A:K68, A:R69, A:N70, A:K71, A:D72, A:N73, A:M74, A:A75, A:A76, A:T77, A:V78, A:K79, A:A80, A:I81, A:E82, A:P85, A:L93, A:L94, A:H95, A:Y96, A:E97, A:D98, A:G99, A:V100, A:K101, A:S102, A:K111, A:V112, A:G113, A:D114, A:K115, A:V116, A:Y117, A:S118, A:G119, A:D120, A:D121, A:V122, A:D123, A:I124, A:K125, A:G127, A:N128, A:T129, A:L130, A:K131, A:L132, A:K133, A:N134, A:I135, A:E145, A:L146, A:K147, A:P148, A:G149, A:K150, A:G151, A:D168, A:N169, A:D170, A:K171, A:Y172, A:L186, A:A187, A:E188, A:N189, A:R190, A:G207, A:K208, A:A209, A:G210, A:R213, A:W214, A:G216, A:F217, A:R218, A:P219, A:T220, A:V221, A:R222, A:G223, A:S224, A:V225, A:M226, A:N227, A:P228, A:N229, A:D230, A:H231, A:P232, A:H233, A:G234, A:G235, A:G236, A:E237, A:G238, A:K239, A:A240, A:P241, A:I242, A:G243, A:R244, A:P245, A:S246, A:P247, A:L248, A:S249, A:P250, A:W251, A:G252, A:K253, A:K254, A:T255, A:L256, A:S291, A:P293, A:S294, A:G295, A:N296, A:D298, A:E299, A:D302, A:G303, A:G304, A:P305, A:G306, A:P307, A:G308, A:E309, A:S312, A:L313, A:I317, A:P320, A:V321, A:T322, A:A323, A:S324, A:Q328, A:G329, A:P330, A:G331, A:P332, A:G333, A:S334, A:D335, A:K336, A:I337, A:V338, A:V339, A:C340, A:N341, A:T342, A:G343, A:E344, A:D345, A:T346, A:R347, A:S348, A:G349, A:N350, A:S351, A:D352, A:P355, A:G356, A:P357, A:G358, A:P359, A:R360, A:G361, A:N362, A:F363, A:E364, A:V365, A:S486, A:Y505	226	0.688
5	A:F407, A:N408, A:K409, A:K411, A:N412, A:V413, A:K414, A:L415	8	0.616
6	A:F388, A:M391, A:N393	3	0.595
7	A:S431, A:S432, A:R433, A:A434, A:T435, A:Y436, A:P437, A:A438	8	0.529

290 residues were found to be located in seven discontinuous B-cell epitopes of the refined vaccine model.

### Molecular docking of the chimeric protein with TLR2

**Figure 5:**
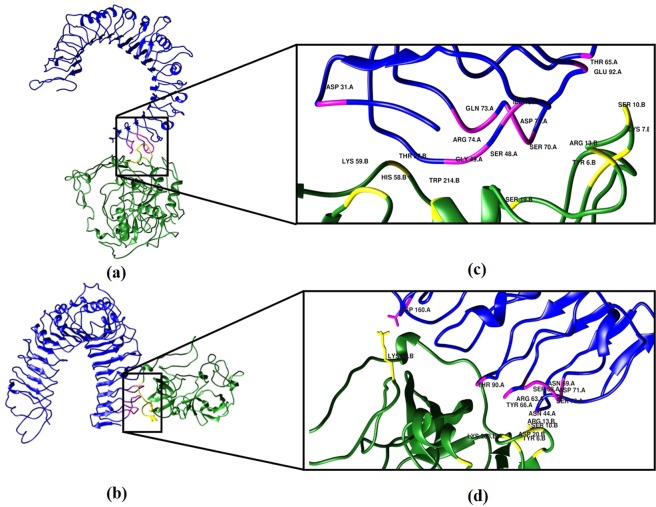
Molecular docking of subunit vaccine with immune receptor (TLR2). (**a**) Docked complexes for Vaccine-TLR2 complex with protein colored sea green, chain A of TLR2 colored medium blue and the interface colored yellow and magenta, and (**b**) adjuvant-TLR2 complex with adjuvant colored sea green, A chain of TLR2 colored medium blue and the interface colored in magenta and yellow. (**c**) Interface active residues for Chimeric vaccine-TLR2 complex with protein active residues colored yellow and TLR2 active residues colored in magenta, and (**d**) Adjuvant-TLR2 complex with protein active residues colored yellow and TLR2 active residues colored magenta.

For initiating an interaction of the vaccine chimera with the TLR2 immune receptor, the binding of protein and hydrophobic contacts on the surface of protein were predicted by the CASTp 3.0 server. A binding pocket was identified as a result which can serve as a possible site for interacting with TLR2 receptor. The molecular surface area of the pocket was 35560.7 Å^2^ with a molecular surface volume of 35804.9 Å^3^, the mouth molecular surface was 5825.3 Å^2^ and the molecular surface circumference sum was calculated to be 6362 Å. For the confirmation of the ability of TLR2 to generate an immune response, it is pertinent to estimate the stability of the designed protein with the docked complex of TLR2 based on its conformation. The comparison of the interaction between TLR2 and protein as well as TLR2 and adjuvant was performed following the docking (data driven) of these two composites. CPORT predicted the provided active interface amino acid residues: T214, T6, L7, T9, A13, G12, L208, A11, SS10, H58, I4, A20, S19, L59 from the adjuvant; and A211, A13, T6, M16, G18, S19, T27, I24, P21, L26, T9, S10, L7, P332, A11, L59, T214 and H58 from the chimeric protein and I46, S45, S68, S48, A71, S70, G52, G53, A74, T65, T66, G92, A44, L50, A31, C60, L28, G49 and T51 from the A chain of TLR2. These active residues were employed to drive the docking protocol. From the HADDOCK results, docked compounds with the highest poses were opted which have minimum intermolecular energies (designed protein-TLR2 complex (−239.2 Kcal/mol), adjuvant-TLR2 complex (−368.1 Kcal/mol) were chosen from those HADDOCK clusters which have the lowest average pairwise backbone RMSD (protein-TLR2 complex (3.9 Å), adjuvant-TLR2 complex (0.8 Å)) at the interface. The relative binding free energies (ΔG) of protein-TLR2 complex (−9.9 Kcal/mol) and adjuvant-TLR2 complex (−9.1 Kcal/mol) are indicative of the linkage of the chimeric protein to the adjuvant sites, thus prompting verified changes that support the stimulation of the TLR2 receptor. Furthermore, a total of 15 hydrogen bonds were formed between the active residues of TLR2 and vaccine whereas, in case of adjuvant-vaccine complex, 14 hydrogen bonds were formed at the interacting surfaces. The analysis of the number of interfacial contacts (IC) per property between the two complexes revealed that predictions (ICs charged-charged: 7, ICs polar-polar: 13, ICs apolar-apolar: 7) for the vaccine and TLR2 complex were more than those predicted for the adjuvant and TLR2 complex (ICs charged-charged: 4, ICs polar-polar:6, ICs apolar-apolar: 6) **(**Fig. 5**)**.

### Codon optimization of the chimeric protein

**Figure 6:**
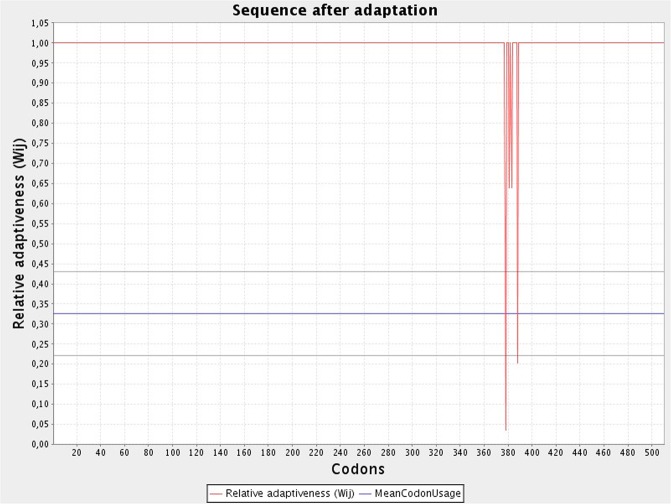
Codon optimization of the final vaccine protein. The CAI of the optimized codon is 0.988 and the average GC content is predicted to be 51.36% as illustrated in the graph.

**Figure 7:**
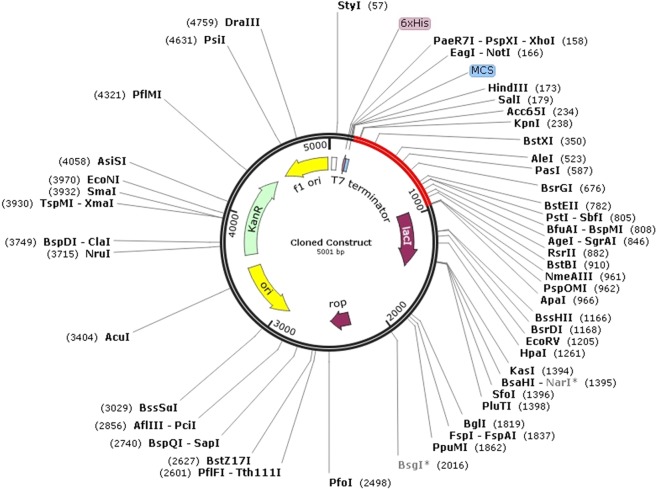
Final protein *in-silico* restriction cloning into the pET28a(+) vector. The red part represents the gene sequence of the designed vaccine protein and the black part indicates the backbone of the *E. coli* vector. The 6xHis tag is situated at the carboxy terminal of the cloned construct.

For the optimization of the codon of the chimeric peptide in *E. coli* (strain K12) for maximum exhibition of protein, the Java Codon Adaptation Tool (JCat) was employed. The codon sequence optimized by the tool had a length of 1500 nucleotides. CAI also known as the Codon Adaptation Index was calculated to be 0.988, with an average GC content of 51.36% for the adapted sequence. Such GC values are indicative of potentially stable expression of the designed vaccine in the selected microbial host **(**Fig. 6**)**. An optimal range for a good GC content lies between 30% to 70%. Furthermore, the designed sequence was integrated into the *E. coli* pET-28a(+) vector for optimal gene expression. This was achieved by incorporating restriction sites followed by the cloning of the genetic sequence into the vector via SnapGene software **(**Fig. 7**)**.

### Characterization of immune profile of the vaccine

**Figure 8:**
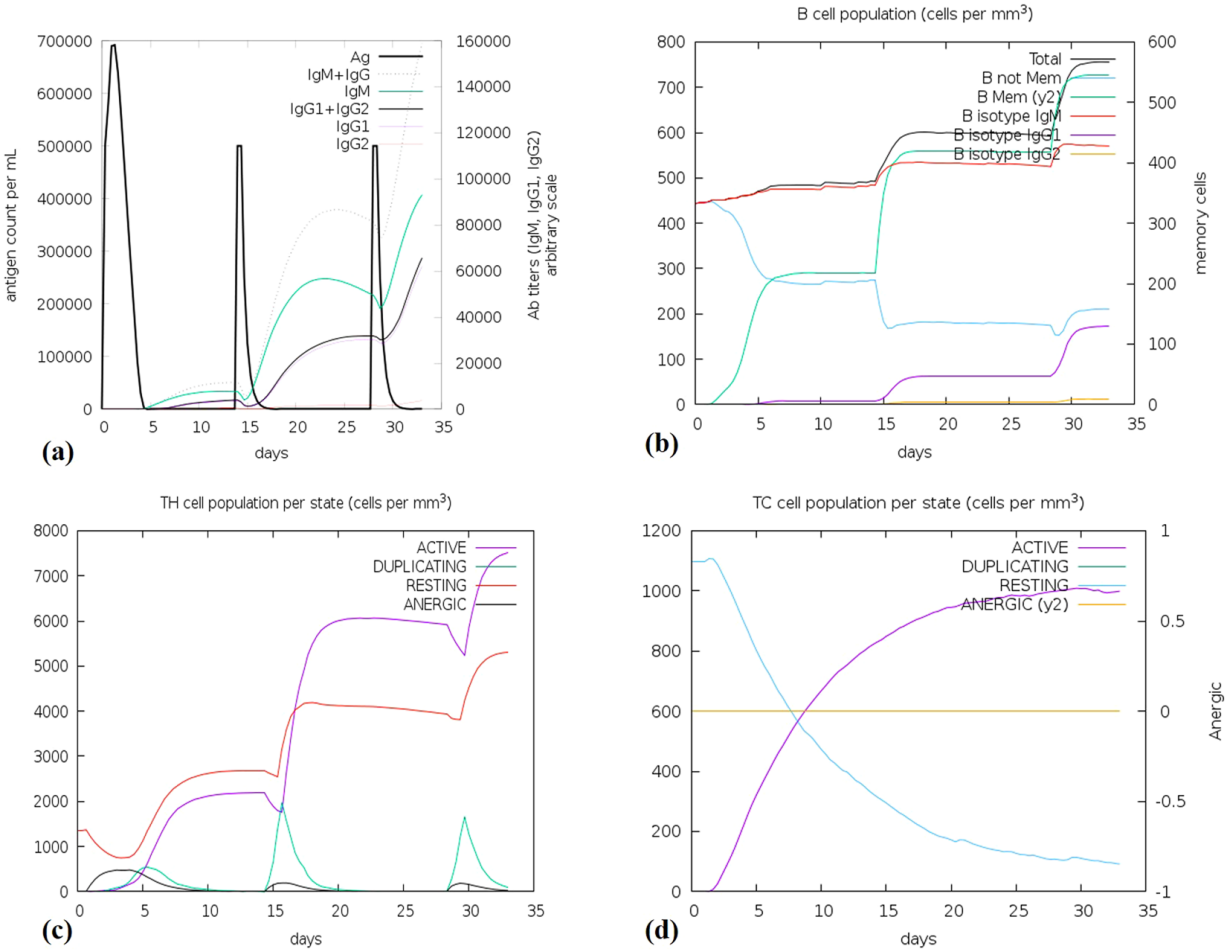
Molecular simulations of the chimeric protein. (**a**) Immunoglobulin production in response to the subsequent antigen injections (black vertical lines); subclasses of immune cells are indicated by colored peaks. (**b**) The changes observed in B-cell populations after given three injections. (**c**) The development of T-helper, and (**d**) T-cytotoxic cell populations per state after the injections. Cells which are not presented with the antigen are indicated by the resting state whereas T cells which show tolerance to the antigen are indicated by the anergic state as a result of repeated exposures.

For the analysis of immune responses created by the final chimeric vaccine construct, the immune simulator C-ImmSim produced such simulations which match with the real responses formed by the immune system. These responses suggest a high spike in the initiation of secondary immune responses. Relatively, high levels of IgM were recorded in the primary response preceded by the surge in B cell populations during the secondary and tertiary responses. Furthermore, with the minimization in the concentration of antigen, the IgG1 + IgG2, IgG + IgM antibodies along with IgM were found to be increased **(**Fig. 8a**)**. The outline illustrates the memory formation in the immune system upon the repeated exposures **(**Fig. 8b**)**. As the memory further strengthens, a projected response was reported in the cytotoxic and helper cell populations **(**Fig. 8c,d**)**.

## Discussion

Enterotoxigenic *B. fragilis* is the most common anaerobic isolate identified in clinical specimens which causes different types of infections related to intestinal and genitourinary tracts^25^. This toxigenic strain has reported to cause severe abscess formation and bacteremia as it is regarded as the only sole infecting microorganism^26^. Anaerobic infections are the major culprits for mortality around the globe and enterotoxigenic *B. fragilis* is frequently associated with such types of infections with a mortality rate of 19%. It is estimated that this rate can go as high as 60% if the infections caused by the *B. fragilis* are left untreated^27^. Moreover, a strong link has been found between the enterotoxigenic *B. fragilis* and the occurrence of colorectal cancer in various murine models^28^. Antibiotics have been used to treat such infections up till now but, with the emergence of antibiotic resistance around the globe, these regimens have lost their efficacies against this toxigenic strain as well. Increased resistance has been documented against clindamycin, cefoxitin, tetracycline, piperacillin-tazobactam, imipenem and even meropenem^29^. Despite the availability of chemotherapeutics for the treatment of various infections, there are always chances of clinical complexities due to drug resistance, mismanagement and risks of complicated infections. Therefore, it is the need of the time to develop an effective therapeutic vaccine for the toxigenic strains of *B. fragilis*. Currently, there is no prophylactic or therapeutic vaccine available for this particular strain, therefore the focus of this study was to devise an apt subunit vaccine as such type of vaccines have reported safe profile and feasibility^30^. Vaccines based on multiple epitopes are unique as they stimulate specific immune responses by eliminating responses generated against the unfavourable epitopes in the antigen^31^. Furthermore, epitopic vaccines have relatively a safer profile and enhanced potency while focusing the immune responses specifically on the selected epitopes^32^.

The main focus of this research was to develop a multi-epitopic subunit vaccine protein using *in-silico* approaches against the enterotoxigenic *B. fragilis*. A total of two proteins were used for generating the protein sequence. These proteins are extracellular proteins which are expressed in the enterotoxigenic strains of the *B. fragilis*. These selected proteins hold the potential of being effective vaccine candidates as they play significant role in the virulence of the subject microorganism^33^.

Immunity to the enterotoxigenic *B. fragilis* is reported to be dependent on both the B and T cells as the polysaccharides produced by the strain are responsible for the mediation of normal immune response^34^. The involvement of TLR particularly TLR2 and TLR4 in generating immunity against *B. fragilis* have been well reported^35^. A study has reported the specificity of TLR2 binding with the bacterial polysaccharide^36^. In another study, it was revealed that the TLR2 receptor along with TLR1 are the main pattern recognition receptors involved in the recognition of *B. fragilis*^37^. As TLR2 is considered to be involved in the immune modulation and cytokine induction in cases related to *B. fragilis* infections, therefore, we initially identified B and T cell epitopes from the two chosen proteins followed by their fusion via suitable linkers for the generation of a multi-epitopic vaccine candidate. Spacer sequences are important in developing vaccines due to their optimum effects^38^. GPGPG and AAY linkers used in previous studies^23,39^ were integrated between the predicted epitopes so as to generate a protein sequence with optimal antigenicity, thus producing a rational vaccine construct. An EAAAK linker was also incorporated in the design for joining the adjuvant with the first predicted B-cell epitope. The involvement of this linker has been reported in designing bifunctional proteins which enhances the fused protein^40^.

The bioinformatic analysis coupled with immunologic analysis indicated the vaccine construct to be full of MHC Class I, MHC Class II possessing increased binding linear and discontinuous B-cell epitopes. The absence of allergenic properties in the final vaccine further affirms its potential as a vaccine candidate. Few studies document poor antigenicity of multi-epitopic vaccine construct and suggest coupling with a potent adjuvant^41^. However, this developed chimera presented satisfactory antigenicity scores in the absence and presence of an adjuvant though; coupling with the probiotic L2 ribosomal protein from *Lactobacillus rhamnosus* GG predicted a higher antigenicity score. The molecular weight of our designed protein is 54 kDa and is analyzed to be soluble which is accordant with its stimulated antigenicity. The theoretical pI of the protein is found to be 9.75, confirming the basic nature of the protein. The instability index of the protein is predicted to be 35.64 which confirms that the protein will be stable whenever expressed which reinforces its putative use as a vaccine model. The aliphatic index of the chimeric vaccine also proved it to be thermostable. All these parameters confirm the thermostability of our designed protein. The secondary and tertiary structure are integral for designing a vaccine candidate. The secondary structure of our chimeric protein is predicted to be consisting of predominately coils (66%) with only 11% disordered residues. As the 3D structure of the protein was refined and improved, preferable properties were observed on Ramachandran plot. It indicates that majority of the residues are present in the favored areas with few residues in the outlier region, thus indicating satisfactory quality of the designed model.

For analyzing the interaction of TLR2 with the designed protein, protein docking was undergone since a probiotic adjuvant was employed in the vaccine construct. The binding energies calculated from the adjuvant and the chimera-adjuvant connections with the immune receptor verified our developed protein to potentially elicit protective immune response.

Immune simulation of the designed vaccine protein revealed results that were harmonious with the immune responses. When the antigen is repeatedly exposed to the antigen, the immune response is enhanced generally. In this case, the development of B and T cells were indicative as well as Helper T cells were also reported to be stimulated. The humoral response was predicted to be generated as the T_H_ production increased.

One of the main ways to validate a designed vaccine protein is to filter it for immunoreactivity^42^ for which expression in an appropriate host is required. For the manufacturing of recombinant proteins, *Escherichia coli* expression systems are the most preferable choice^43^. For expressing our designed vaccine construct in *E. coli* (strain K12), optimization of the codon was performed. The codon adaptability index (0.988) and the GC content (51.36%) were desirable for the maximum expression of the protein in the microorganism.

## Conclusion

For effective elimination of enterotoxigenic *B. fragilis*, a novel vaccine is essential as antibiotic resistance against the pathogen is increasing day by day. In this study, *in-silico* tools were employed for constructing a potential vaccine that codes for multiple B cell and T cell epitopes. The proteins selected for this study are exclusively expressed in the toxigenic *B. fragilis* thereby, proving to be putative candidates for the elimination of the pathogen.

## Methodology

### Selection of protein sequences for vaccine designing

A total of two proteins were selected for the preparation of the vaccine. These proteins are expressed in the Enterotoxigenic *B. fragilis* strains. The pathogenicity island gives rise to enterotoxin Fragilysin and a second metalloprotease (MP II) proteins whose combined action is responsible for the virulence of the subject pathogen. The complete amino acid sequences of proteins WP_005797262.1 and WP_005797263.1 were retrieved from NCBI database (https://www.ncbi.nlm.nih.gov/) in FASTA format. Further, SignalP 4.1 server was used for the analysis of the signal peptides (http://www.cbs.dtu.dk/services/SignalP/) in order to differentiate the secretory and non-secretory proteins. It also indicates the positioning of cleavage sites in the proteins by making use of various artificial neural networks^44^. Subcellular-localization of the proteins were checked on Vaxign (http://www.violinet.org/vaxign/) which is a reverse vaccinology-based pipeline that predicts various subcellular locations by utilizing PSORTb 2.0 which is reported to have a measured prediction of 96%^45^. To achieve accuracy for the prediction of localization, the proteins were also subjected to DeepLoc 1.0 (http://www.cbs.dtu.dk/services/DeepLoc/) that gives the subcellular localization of eukaryotic proteins with the assistance of deep neural networks^46^.

### Identification of linear B-cell epitopes

Linear B-cell epitopes serve an integral role in the process of vaccine designing and production. These epitopes are characterized as antigenic determinants which are identified by the immune system. Moreover, the B lymphocytes bind to these specific pieces of the antigen and evoke an immune response^47^. For this study, the linear B cell epitopes were mainly predicted by BepiPred-2.0 web server (http://www.cbs.dtu.dk/services/BepiPred/). It works on the principle of a forest-based algorithm that is built on annotated epitopes from the antigen-antibody structures of proteins. The server generates reliable prediction of epitopes as compared to other available servers as it takes account of the solved 3D structures of proteins and database of linear epitopes collected from the IEDB database^48^. Multiple tools for forecasting the B cell epitopes were utilized as this strategy helps to achieve accurate results. Next, the protein sequences were subjected to BCPred (http://ailab.ist.psu.edu/bcpred/) which works for linear epitope prediction. This server functions on string kernels for predicting the antigenic epitopes by fusing the tri-peptide similarity and propensity scores. The AUC value for the server lies within an acceptable range^49^.

### Estimation of cytotoxic T lymphocytes (CTL) epitopes

NetCTL 1.2 server (http://www.cbs.dtu.dk/services/NetCTL/) was employed for the projection of cytotoxic T lymphocytes for the selected proteins. NetCTL server predicts the CTLs by integrating the estimation of three vital processes such as MHC class I binding peptides, proteasomal C-terminal cleavage and transporter that is associated with antigen processing (TAP) transporter efficiency. With the help of this web portal, CTL epitopes can be predicted for 12 MHC class I supertypes but, for this study, only the A1 supertype was utilized. NetCTL server predicts the outcomes on the basis of artificial neural networks and a weight matrix is generated which predicts the TAP transporter efficiency. Default settings were used (threshold, 0.75) for the estimation of CTL epitopes^50^.

### Prediction of helper T cells (HTL) epitopes

The helper T cells 15-mer epitopes for the selected two protein sequences were predicted by using the NetMHCII 2.2 server (http://www.cbs.dtu.dk/services/NetMHCII/). The NetMHCII server utilizes the artificial neuron networks for predicting the linkage of peptides to the human alleles HLA-DR, HLA-DQ and HLA-DP. Moreover, the server predicts the MHC II epitopes on the basis of receptor affinity which is usually inferred from the IC_50_ values. According to standards, high affinity peptides fall within the range of <50 nM IC_50_ values^51^.

### Conservation of proteins and epitopes in related *Bacteroides*

To assure that the selected proteins chosen for the design of vaccine construct are exclusively expressed only in the toxigenic strains of *B. fragilis*, a BLAST search was undertaken on the UniProt database for procuring the rank of identities among the related relatives of the chosen proteins. Furthermore, the extent of conservation of the selected epitopes was also estimated following the multiple sequence alignment of the homologous proteins in related *Bacteroides*. The species opted for the comparison enclosed the nontoxigenic *B. fragilis* i.e. *B. fragilis YCH46*, *B. fragilis 638 R*, *B. fragilis NCTC 9343* to assure that the designed vaccine will only target the toxigenic strains and will not cause any harm to the commensal strains of the same species. *Bacteroides distasonis*, *Bacteroides ovatus*, *Bacteroides thetaiotamicron*, *Bacteroides vulgatus* and *Bacteroides uniformis* candidates were selected on the basis of their high contribution in the anaerobic infections^52^.

### Assemblage of multi-epitopic vaccine candidate sequence

The putative vaccine candidate sequence was devised by combining the high scoring epitopes of B cells, CTLs and high affinity binding HTLs epitopes. B cell epitopes were predicted simultaneously by BepiPred and BCPred servers. To enhance the immunogenicity of the protein vaccine, a 50 s ribosomal protein L2 of a probiotic *Lactobacillus rhamnosus* GG (Accession no. AXI95322.1) was preferred as an adjuvant whose sequence was derived from the UniProt database (http://www.uniprot.org/). The adjuvant was attached to the first B-cell epitope through an EAAAK linker at the N terminal of the sequence whereas, the remaining B-cell and HTL epitopes were connected to each other via GPGPG linkers. AAY linkers were used for joining the CTLs epitopes and a 6x His tag was added at the C terminal for protein identification and purification.

### Evaluation of antigenicity and allergenicity of the protein

In order to predict the antigenicity of the chimeric construct, VaxiJen v2.0 and ANTIGENPro servers were utilized. VaxiJen v2.0 is a feely accessible server (http://www.ddgpharmfac.net/vaxijen/VaxiJen/VaxiJen.html) which functions on the auto and cross variance (ACC) transformation of proteins and convert them into uniform vectors of principal amino acid properties. It generates the antigenicity of the proteins without involving any alignment and focuses on the physiochemical properties of the selected candidate^53^. Another server was used to guess the antigenic nature of the designed peptide. ANTIGENPro (http://scratch.proteomics.ics.uci.edu/) is a free server which employs a specific microarray data for the calculation of protein antigenicity index. On the basis of cross-validation experiments, the accuracy of the server was reported to be 76%^54^.

The allergenicity of the multi-epitopic vaccine was predicted by AllerTOP v2.0 and AllergenFP. AllerTOP v2.0 (http://www.ddg-pharmfac.net/AllerTOP) is a freely accessible server that makes use of the machine learning methods such as amino acid E-descriptors, auto and cross variance transformation and the k nearest neighbors, for classifying the allergens. At five-fold cross validation, this server has an accuracy of 85.3%^55^. AllergenFP is another online server which utilizes a descriptor-based fingerprint approach to differentiate between the antigens and allergens. This approach is alignment free and consists of four main steps. As a result, an accuracy of 88% was achieved with a Mathews correlation coefficient of 0.759^56^.

### Analysis of solubility and physiochemical properties

To evaluate the solubility of the designed vaccine sequence, PROSO II online server (http://mbiljj45.bio.med.uni-muenchen.de:8888/prosoII/prosoII.seam) was used. The server employs a classifier that has the capability of identifying minute differences between the soluble and insoluble proteins stored in TargetDB and PDB. Evaluation at 10-fold cross validation yields an accuracy of 71% with an area of 0.785 being present under the ROC curve^57^. Furthermore, the designed protein sequence was assessed for a number of physiochemical properties by using the ProtParam (http://web.expasy.org/protparam/) server^58^.

### Extrapolation of secondary structure of the construct

PSIPRED and RaptorX were used for the generating the secondary structure of the vaccine protein. PSIPRED ((http://bioinf.cs.ucl.ac.uk/psipred/) is a freely accessible online server. It utilizes the position specific iterated BLAST for the identification and selection of those sequences that show significant similarity to the designed vaccine. Overall, PSIPRED 3.2 has a Q3 score of 81.6%^59^. Next, to predict the secondary structure, another webserver RaptorX was employed (http://raptorx.uchicago.edu/StructurePropertyPred/predict/). It is an alignment free server that engages an innovative machine learning method DeepCNF to provide the secondary structure, solvent accessibility and disordered regions at once with satisfactory accuracy^60^.

### Assessment of tertiary structure of the protein

The final multi-epitopic vaccine sequence was then subjected to I-TASSER server for homology modelling (https://zhanglab.ccmb.med.umich.edu/I-TASSER/). I-TASSER (Iterative Threading ASSEmbly Refinement) is a best ranked server which is used for the generation of automated protein structures and prediction. Upon submission of an amino acid sequence, I-TASSER works to design a 3D atomic model by utilizing the multiple threading alignments and iterative structural assembly simulations^61^. Another online server Phyre2 (http://www.sbg.bio.ic.ac.uk/phyre2/) was also employed for the homology modelling of the designed peptide. This server uses latest detection methods to construct three dimensional structures and performs the prediction of ligand binding sites^62^.

### Refinement of the tertiary structure of protein

The three dimensional protein model obtained via the I-TASSER server, was further subjected to a two-step refinement procedure by using the ModeRefiner (https://zhanglab.ccmb.med.umich.edu/ModRefiner/) followed by the GalaxyRefine (http://galaxy.seoklab.org/cgi-bin/submit.cgi?type=REFINE) online servers. ModeRefiner works on a dual step atomic energy level minimization process which constructs and refines the given protein structures from Cα traces. This procedure assists in enhancing the local and global structures with accurate and reliable results^63^. Next, GalaxyRefine was implemented for refining the protein structure. This server utilizes a refinement method that establishes side chains and repack them, thus ultimately achieving an overall relaxation of the structure by integrating dynamic simulations. Various experimentations have regarded this server as one of the best refinement servers which improves the quality of both the local and global structures^64^.

### Validation of the model stability

For the evaluation of the stability of the devised model, its validation is integral as it detects potential errors that might be present in the predicted 3D protein models. For this purpose, ProSA-web server (https://prosa.services.came.sbg.ac.at/prosa.php) was used initially which is frequently used for the validation of tertiary structure of the protein. It calculates the overall quality score with regards to the context of all the known proteins structures. The erroneous parts of the structures are then displayed in the server’s molecular viewer^65^. Withal, ERRAT (http://services.mbi.ucla.edu/ERRAT/) was used which analyzes the non-bonded atom to atom interactions while comparing them to high resolution crystallography structures. For the generation of the Ramachandran plot, MolProbity and RAMAPGAE servers were used. Ramachandran plot is a method which is used to visualize the energetically allowed and disallowed dihedral angles constituting the psi (ψ) and phi (φ) of an amino acid. This calculation is mainly performed on the basis of the van der Waal radius of the side chains. MolProbity (http://molprobity.biochem.duke.edu/) is an all atom structure validation online server that offers Ramachandran analysis^66^. RAMPAGE (http://mordred.bioc.cam.ac.uk/~rapper/rampage.php) is another freely accessible server that integrates the PROCHECK principle for the validation of the protein model through the application of Ramachandran plot and divides the Glycine and Proline residues plot^67^.

### Prediction of discontinuous B-cell epitopes

For the prediction of discontinuous epitopes of B cells for the validated protein model, ElliPro (http://tools.iedb.org/ellipro/) was utilized. Based on few reports, it is estimated that more than 90% of the epitopes of B cells are discontinuous. The freely accessible online server integrates mainly three algorithms to stabilize the protein shape followed by the calculation of the residue protrusion index. This in return leads towards the clustering of neighboring residues on the basis of their PI values. ElliPro is one of the best servers of their kind with an estimated AUC score of 0.732 which is a significant score for prediction^68^.

### Molecular docking of the chimeric protein with TLR2

An appropriate immune response can only be produced when an antigenic molecule gets to interact with a specific immune receptor in the host. For this vaccine construct, TLR2 receptor was found to be an apt immune receptor whose binding pocket and cavities were identified by utilizing the CASTp server (http://sts.bioe.uic.edu/castp/). CASTp is an efficient online server which identifies and measures the surface accessible binding pockets and provides information related to the inner unapproachable cavities for the given proteins^69^.

Next in the process, HADDOCK 2.2 web server was employed for the molecular docking of the multi-epitopic chimeric vaccine construct with the TLR2 receptor whose structure was taken from PDB (https://www.rcsb.org). Docking a peptide with a specific immune receptor helps to analyze the interaction between the ligand and the receptor which steers towards the formation of an immune response. TLR2 has found to confer protective immunity in intestinal diseases specifically in inflammatory bowel disease and colorectal cancer^70–72^. For the molecular docking, data driven docking of the designed protein with the TLR2 and the adjuvant with the TLR2 was undertaken. The adjuvant selected is a 50 s ribosomal protein, L2 (Accession ID. AXI95322.1) whose structure was predicted by using SWISS-MODEL (https://swissmodel.expasy.org/). This protein has the ability to prompt the TLR2 receptor, thus serving as a TLR2 agonist^73^.

For accurately predicting which residues would be involved in the docking interactions, CPORT (https://milou.science.uu.nl/services/CPORT/) was employed^74^. It predicted the active residues at the interface of the vaccine protein including the adjuvant, adjuvant and the TLR2. After collecting the active and passive residues for the structures involved, HADDOCK 2.2 (http://haddock.science.uu.nl/services/HADDOCK2.2) was used to execute the docking simulations for the vaccine protein-TLR2 and adjuvant-TLR2 composites^75^. The High Ambiguity Driven DOCKing (HADDOCK) is a python-based server which employs the crystallography systems for structure collection. The server works in a sequential manner where at first, the structures are properly oriented and their subsequent docking calculations are executed. Afterwards, the server confirms the conclusive structures and invokes a molecular dynamics simulation stage. The inter and intra molecular energies of the provided structures are predicted by utilizing the van der Waal and electrostatic energy terms^76^. The binding affinities of the complexes were predicted by employing the PRODIGY server (https://nestor.science.uu.nl/prodigy/)^77^.

### Codon optimization of the chimeric protein

In order to incorporate and express the designed multi-epitopic construct in a selected expression vector, the reverse translation and codon optimization of the protein sequence must be executed. For this purpose, Java Codon Adaptation Tool (JCAT), an online web server was used. The final construct was expressed in *E. coli* (strain K12) as the native host *B. fragilis* differs from this strain. While using JCAT (http://www.prodoric.de/JCat), three of the provided additional options were availed for avoiding the termination of the rho-independent transcription, binding site of the prokaryotic ribosome and cleavage sites of restriction enzymes. The output received from the tool consists of a codon adaptation index (CAI) and percentage of GC content that is indicative of the expression levels of the protein. The codon usage biases are indicated by the CAI where a score of 1 is considered to be ideal whereas scores greater than 0.8 fall in the good and acceptable category. The GC content of the protein sequence should be within the range of 30–70% as scores which fall outside this range are indicative of unfavorable effects on the transcription and translation performances^78^. The designed vaccine sequence was transformed into a suitable host vector pET-28a(+) by employing the SnapGene software.

### Characterization of immune profile of the construct

For analyses of the immune responses of the final vaccine candidate, immune simulations were executed via the C-ImmSim server (http://150.146.2.1/C-IMMSIM/index.php). This online server functions on the basis of a position specific scoring matrix (PSSM) for the foretelling of immunogenic epitopes and immune interactions. For the vaccine candidate, all the default simulation parameters were used with time steps specified at 1, 42 and 84. Thereby, three injections were given at a time^79^.

## Data Availability

All data generated or analyzed during the study are included in the submitted manuscript. The sequences of the protein analyzed can be retrieved from UniProt database (uniport.org) using their accession numbers.
